# HPV-Related Skin Phenotypes in Patients with Inborn Errors of Immunity

**DOI:** 10.3390/pathogens11080857

**Published:** 2022-07-29

**Authors:** Assiya El Kettani, Fatima Ailal, Jalila El Bakkouri, Khalid Zerouali, Vivien Béziat, Emmanuelle Jouanguy, Jean-Laurent Casanova, Ahmed Aziz Bousfiha

**Affiliations:** 1 Laboratory of Clinical Immunology, Inflammation and Allergy LICIA, Faculty of Medicine and Pharmacy, Hassan II University, Casablanca 20250, Morocco; drailalfatima@gmail.com (F.A.); jalilaelbakkouri@gmail.com (J.E.B.); khalid.zerouali2000@gmail.com (K.Z.); profbousfiha@gmail.com (A.A.B.); 2 Laboratory of Bacteriology, Virology and Hospital Hygiene, Ibn Rochd University Hospital, Casablanca 20250, Morocco; 3 Laboratory of Bacteriology and Virology, Faculty of Medicine and Pharmacy, Hassan II University, Casablanca 20250, Morocco; 4 Clinical Immunology and Infectious Pediatrics Department, Abderrahim Harouchi Hospital, Ibn Rochd University Hospital, Casablanca 20250, Morocco; 5 Immunology Laboratory, Ibn Rochd University Hospital, Casablanca 20250, Morocco; 6 Laboratory of Human Genetics of Infectious Diseases, Necker Branch, Institut National de la Santé et de la Recherche Médicale (INSERM), 75015 Paris, France; vivien.beziat@inserm.fr (V.B.); emmanuelle.jouanguy@inserm.fr (E.J.); casanova@mail.rockefeller.edu (J.-L.C.); 7 Laboratory of Human Genetics of Infectious Diseases, Rockefeller Branch, Rockefeller University, New York, NY 10065, USA; 8 Howard Hughes Medical Institute, Chevy Chase, MD 20815, USA

**Keywords:** HPV, skin, inborn errors of immunity

## Abstract

Patients with inborn errors of immunity (IEI) are prone to develop infections, either due to a broad spectrum of pathogens or to only one microbe. Since skin is a major barrier tissue, cutaneous infections are among the most prevalent in patients with IEI due to high exposures to many microbes. In the general population, human papillomaviruses (HPVs) cause asymptomatic or self-healing infections, but, in patients with IEI, unusual clinical expression of HPV infection is observed ranging from epidermodysplasia verruciformis (EV) (a rare disease due to β-HPVs) to profuse, persistent, and recalcitrant warts (due to α-, γ-, and μ-HPVs) or even tree man syndrome (due to HPV2). Mutations in EVER1, EVER2, and CIB1 are associated with EV phenotype; GATA2, CXCR4, and DOCK8 mutations are typically associated with extensive HPV infections, but there are several other IEI that are less frequently associated with severe HPV lesions. In this review, we describe clinical, immunological, and genetic patterns of IEI related to severe HPV cutaneous infections and propose an algorithm for diagnosis of IEI with severe warts associated, or not, with lymphopenia.

## 1. Introduction

Human papillomaviruses (HPVs) are DNA viruses with a specific tropism to keratinocytes, which are the main component of stratified epithelia, including skin, genital, and laryngeal mucosa. There are more than 200 different genotypes of HPVs classified in five (α-, β-, γ-, μ-, and ν-) genera. HPV subtypes of all genera infect the skin, and only some HPVs of α-genus infect the mucosal epithelia. Some α- and β-HPV types are oncogenic and are associated with benign genital condyloma, cervical and anogenital cancers, and non-melanoma skin cancers, respectively [1,2].

In the general population, HPVs cause asymptomatic or self-healing infections, with spontaneous clearances reported: 23% at 2 months and 66% by 2 years [3,4]. The transmission is from skin-to-skin or mucous-to-mucous contact. Seroprevalence is variable, depending on the HPV genus, age, and screening policy of each country. However, it is estimated to be <40% and 20–65% for oncogenic α-HPV and β-HPVs, respectively. Cervical cancer is the main clinical concern following HPV infection, as it is the fourth most frequent female cancer, with a death rate around 7.5%. In addition, more than 85% of deaths due to cervical cancer are in developing countries. The incidence of cutaneous warts varies with age, with a range from 1 to 12% in the adult general population, but could be over 24% in school age children [5]. 

Inborn errors of immunity (IEI) are characterized by an impaired immune response, affecting tissue-intrinsic immunity that is either, innate, adaptive, or both. IEI could be associated with higher susceptibility to infections, auto-inflammation, and/or autoimmunity. Unusual clinical expression of HPV infection is frequently observed in patients with IEI. The spectrum of the clinical phenotype is large from epidermodysplasis verruciformis (EV) (a rare disease due to β-HPV) to profuse, persistent and recalcitrant warts (due to α-, γ-, and μ-genera) [6]. Very rare individuals develop tree man syndrome (TMS) due to HPV2 [7].

There are some published reviews and case reports that describe clinical, immunological, and genetic patterns of IEI related to severe HPV cutaneous infections, but there are too many aspects of these issues that are still unknown and are being discovered continuously. Here, we present an up-to-date review of the major clinical, immunological, and genetic patterns of IEI related to severe HPV cutaneous infections, and we propose an algorithm for diagnosis of IEI with severe warts in order to help clinicians who may encounter patients with recurrent and recalcitrant warts due to an underlying inherited immunodeficiency.

## 2. Clinical Phenotypes

Depending on the HPV genera, there are different characteristics of HPV skin lesions. Macroscopy and histology analyses could help with an appropriate diagnosis. 

### 2.1. Epidermodysplasia Verruciformis

**Figure 1:**
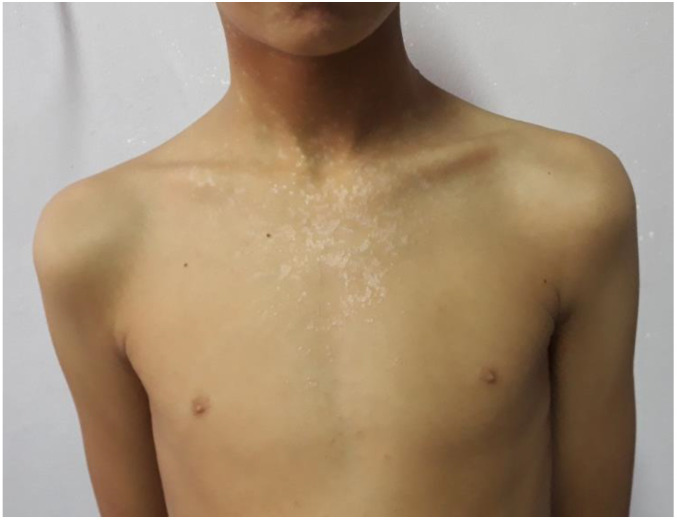
Epidermodysplasia verruciformis lesions initially localized on the face, and then generalized to the neck and the trunk in a 12-year-old male patient with STK4 deficiency.

With less than 250 cases reported worldwide, epidermodysplasia verruciformis (EV) is a rare disease that appears at young ages: infancy (7.5% of cases), childhood (61.5% of cases), and adolescence (22% of cases). Lesions are characterized by progressive onset hyperpigmented or achromic flat verrucous lesions, irregular patches of a reddish-brown color, keratotic seborrheic lesions, and pityriasis versicolor-like macules. The lesions are found mainly on sun-exposed areas, such as the face, trunk, neck, forearms, hands, and feet (Figure 1). Although various genotypes of β-HPVs are detected in EV lesions, HPV5 and -8 are found in 80% of cases. Histologic features of an EV lesion are characterized by a flat wart and showing mild to moderate hyperkeratosis, hypergranulosis, and acanthosis of the epidermis. The keratinocytes in the upper layer of the epidermis are enlarged and exhibit a vacuolated nucleus and a pale blue-gray color [8].

EV can be isolated (typical EV) or syndromic (atypical EV) associated with other clinical manifestations, infectious, or not. [9]. Among 40–50-year-old patients, 30 to 60% of EV patients develop non-melanoma skin cancer, particularly squamous cell carcinoma, occurring in sun-exposed areas. People with black skin have a much lower incidence of skin cancer. Most squamous cell carcinomas remain localized. Metastases are not frequent [8,10].

### 2.2. Profuse Warts (PWs)

**Figure 2:**
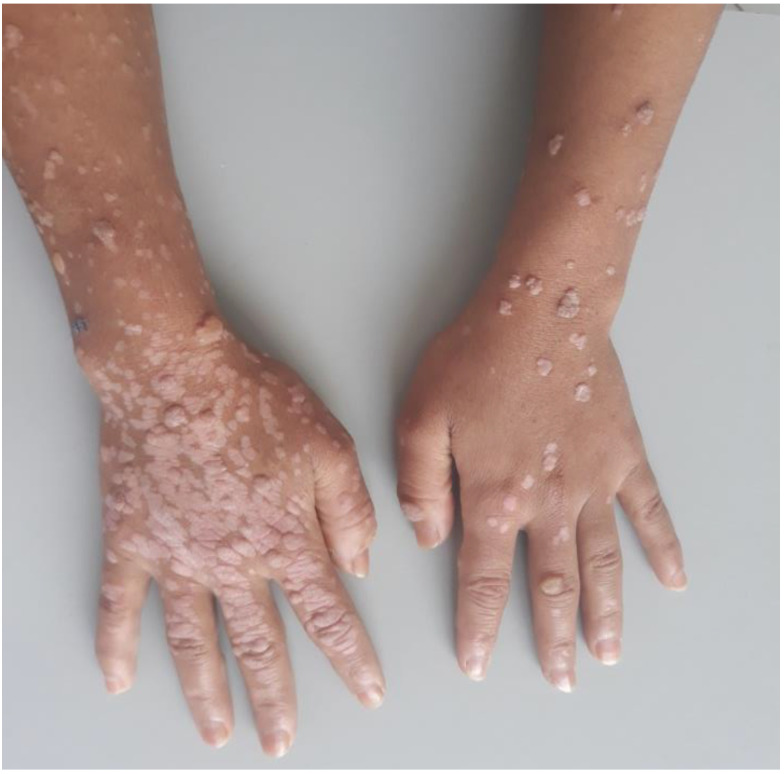
Profuse cauliflower and flat warts in a 26-year-old female patient with GATA2 deficiency (DCML syndrome).

Profuse warts (PWs) are defined as more than 20 lesions in more than one area of the body. If they do not disappear after 6 months of treatment, they are also classified as recalcitrant [11]. PW cauliflower-like papules have a rough, hyperkeratotic surface but they can be flat depending on the HPV involved (Figure 2). PWs are the consequence of an infection with α- or γ-HPV, and less frequently with μ- and ν-HPVs [12]. Histologic analyses of PWs have shown markedly papillomatous epidermis with hypergranulomatosis and overlying tiers of parakeratosis. The upper epidermis may contain large pink inclusions, particularly in cases arising on acral skin. Other lesions have shown smaller basophilic granules. Classically, in the upper epidermis, koilocytes or vacuolated keratinocytes which have a small shrunken nucleus surrounded by a perinuclear halos are observed [13].

### 2.3. Tree Man Syndrome

**Figure 3:**
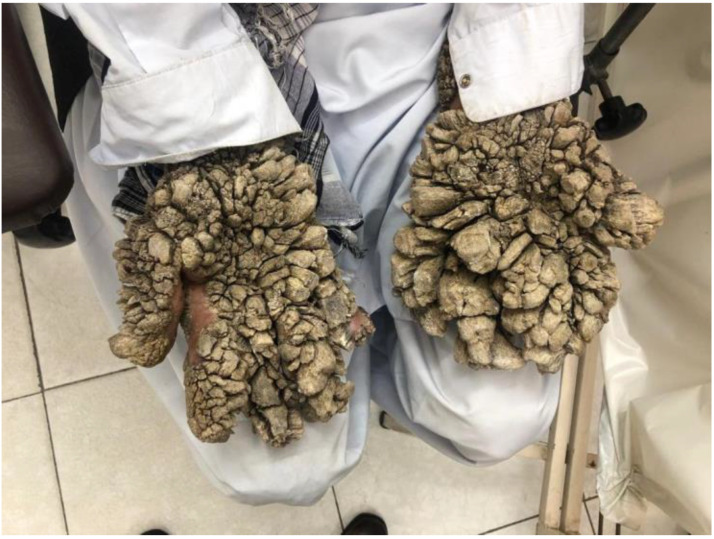
Tree man syndrome giant horns from an HPV2-driven multifocal benign epithelial tumor overexpressing viral oncogenes in the epidermis basal layer in a 30-year-old male patient with *CD28* deficiency [7].

In exceptional cases, the warts can also transform into exophytic cutaneous lesions and giant horns, resulting in tree man syndrome [7]. TMS presents with the most extensive warts developing into cutaneous horns, which can be giant and generalized. These lesions start as cutaneous warts, slowly spreading over the hands and feet before transforming into cutaneous horns, characteristic of the TMS phenotype (Figure 3). This condition is extremely rare, with less than 10 cases reported so far. All cases were sporadic with no family history. Due to the paucity of reported cases, it is unclear whether these lesions in TMS have malignant potential [7].

## 3. Immunological Phenotypes and Inborn Errors of Immunity

### 3.1. No immunological Phenotype in Blood (Skin-Intrinsic Immunity Disorder)

Isolated EV is due to autosomal recessive (AR) mutations in *TMC6* and *TMC8*, which encode EVER1 and EVER2, two endoplasmic reticulum plasma membrane proteins, respectively, and in *CIB1*, which encodes calcium and integrin binding protein [9,14] (Table 1). Patients with isolated EV did not show any major leukocyte abnormalities, neither quantitative nor qualitative, in terms of proliferation or antibodies production. The HPV proteins, E5 and E8, targeted the EVER1–EVER2–CIB1 complex, strongly suggesting that this complex is acting as a restriction factor to HPVs in keratinocytes. In terms of the physiological mechanism, the dominant hypothesis is that isolated EV is the consequence of IEI affecting the keratinocyte-intrinsic immune response [14]. 

### 3.2. Immunological Phenotype with Qualitative or/and Quantitative T Cells Defects Only

In contrast to isolated EV, syndromic EV is related to IEI affecting T cells. Some of these IEI are also associated with PW phenotype. For some of them, warts are a major clinical symptom (Table 2) [6]. For instance, in AR DOCK8 deficiency, warts were reported in >40% of patients that were characterized by T and NK cell lymphopenia, and some patients developed α-HPV-induced malignancies [15]. Furthermore, AR mutations in the serine/threonine kinase 4 (*STK4*) gene are primarily characterized by a reduced amount and survival of circulating naïve T cells. Progressive CD4 T cell lymphopenia with profoundly low naïve CD4 T cell counts is hallmark, while CD8 T cells and NK cells are within normal range. T cell proliferation responses to both antigens and mitogens are markedly impaired. B cell counts are mildly low with hypergammaglobulinemia of IgG and variable increases in IgA and IgE [16].

More recently, patients with CARMIL2 and CD28 deficiencies were associated with HPV susceptibility [7,17]. These IEI both affect the CD28 signaling pathway, which is the major costimulatory pathway of TCR. Patients with CARMIL2 deficiency developed disseminated warts among other infectious manifestations, and they also had decreased memory B cells [17], whereas CD28 deficiency was associated with PW only. Interestingly, one of the CD28 patients developed TMS [7].

### 3.3. Immunological Phenotype with Several Impaired Leukocyte Subsets

This category includes warts, hypogammaglobulinemia, infections, myelokathexis (WHIM) syndrome, and classical CID and SCID syndromes (Table 3). The warts are also due to α-HPV and the immunological phenotypes of these diseases are variable but qualitative or/and quantitative T cell defects are common to all of them [24]. For example, in WHIM syndrome, between 60 and 80% of patients develop warts after α-HPV infection, and about 16% of these patients develop HPV-related cancers. This disease is associated with mutations in the *CXCR4* gene, encoding a chemokine receptor. The immunological phenotype is characterized by neutropenia, low counts of dendritic cells (DC), memory B cells, and naïve CD4^+^ and CD8+ T cells [26]. In GATA2 haploinsufficiency, α-HPV infections occur in more than 50% of the cases, and genital cancers are frequent. Low monocyte, DC, B cell, CD4^+^ T cell, and NK cell counts are the most common immunological features of the patients [18,24].

## 4. Warts and IEI: Diagnostic Strategy

**Figure 4:**
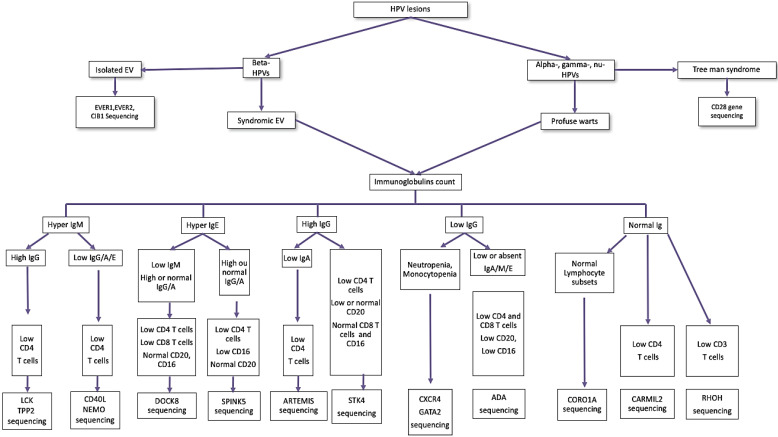
Algorithm for laboratory testing orientation to diagnosis IEI with severe warts and lymphopenia. DOCK8, dedicator of cytokinesis 8; EV, epidermodysplasia verruciformis; ADA, adenosine desaminase severe combined immunodeficiency; NEMO, nuclear factor κB essential modulator deficiency; TPP2, tripeptidyl peptidase 2; LCK, lymphocyte-specific protein tyrosine kinase; SPINK5, serine peptidase inhibitor Kazal type 5; STK4, serine/threonine kinase 4; CXCR4, C-X-C motif chemokine receptor 4; CORO1A, coronin 1A, CARMIL2, capping protein regulator and myosin 1 linker 2, RHOH, Ras homolog family member H.

When an HPV-related clinical manifestation is severe, meaning profuse, chronic or recalcitrant and resistant to treatment, an IEI should be suspected especially if there are other infections, atopy, autoimmunity, or malignancy. Together with familial and patient history and physical examination, a guided differential diagnosis hypothesis should be formulated. Afterwards, focused laboratory testing should be investigated starting with immunoglobulin levels, T cell counts, and T cell subpopulation counts [29]. In Figure 4, we propose an algorithm for laboratory testing orientation for diagnosis of IEI related to HPV susceptibility, with or without impaired leukocyte populations.

## 5. Conclusions

HPV skin lesions are a common symptom during infancy to childhood. Although recalcitrant warts, or even EV, are a rare clinical manifestation, physicians, including dermatologists and pediatricians, should consider IEI for a patient with recurrent or disseminated HPV skin lesions. The diagnosis strategy is crucial for a prompt and appropriate treatment of those patients. Furthermore, investigations of patients with EV or PW will increase our understanding of skin-intrinsic host immunity against HPVs.

## Figures and Tables

**Table 1:** Etiologies and immunological phenotypes of isolated EV.

HPV Phenotype	Gene/protein (Mode of Inheritance)	Clinical Phenotypes	T Cell Counts	T Function	Other Immunological Features	Reference
Isolated EV	*TMC6*/EVER1 (AR)	EV	Normal	Normal	None	[9,14]
	*TMC8*/EVER2 (AR)	EV	Normal with slightly high proportions for skin-homing subsets	Normal	None	
	CIB1 (AR)	EV	Normal	Normal	None	

AR, autosomal recessive; EV, epidermodysplasia verruciformis; TMC6, transmembrane channel-like protein 6; TMC8, transmembrane channel-like protein 8.

**Table 2:** Etiologies, clinical phenotypes, and immunological phenotypes of warts associated with IEI with qualitative or/and quantitative T cell defects.

HPV Phenotype	Gene/Protein (Mode of Inheritance)	Other Clinical Phenotypes	T Cell Counts	T Function	Other Immunological Features	Reference
Syndromic EV	RHOH (AR)	Cutaneous viral infections, bronchopulmonary disease, Burkitt lymphoma	Low naïve CD4+ Tc, high memory CD4+ and CD8+ Tc counts, low proportions of skin-homing Tc subsets	Mildly impaired antigen-induced Tc proliferation, no anti-CD3-induced proliferation	-	[18,19]
Syndromic EV or profuse warts	STK4 (AR)	Bacterial, candida infections, EBV lymphoproliferation, lymphoma, congenital heart disease	Low Tc Low terminal differentiated effector memory cells Low naïve Tc	Poor proliferation Impaired mitogen (PHA, PMA/ ionomycin)- and antigen (candida, tetanus toxoid, tuberculin)- induced proliferation	Intermittent neutropenia, autoimmune cytopenia, low Bc	[16,20,21]
Syndromic EV or profuse warts	DOCK8 (AR)	Cutaneous staphylococcal and viral infections, severe eczema, severe atopy	Low Tc CD4+	Poor production of antiviral cytokines (TNFα, IFNγ)	Hyper IgE, hyper eosinophilia Low IgM	[15,22]
Syndromic EV or chronic warts	CORO1A (AR)	Severe varicella, molluscum contagiosum and aggressive EBV infection	Low Tc	-	Defective number and/or cytolytic activity of NK cells, hypogammaglobulinemia, and defective antibody responses	[18,23]
Syndromic EV	RASGRP1 (AR)	Recurrent pneumonia, herpes virus infections, EBV-associated lymphoma	Low Tc	Tc: poor activation, proliferation, motility	Increased IgA, Bc: poor activation, proliferation, motility	[6,18,24]
Syndromic EV	LCK (AR)	Failure to thrive, severe diarrhea, opportunistic infections	Low CD4+ Low Tregs, restricted Tc repertoire	Poor TCR signaling	Autoimmunity, high IgM	[18,20,24]
Syndromic EV	TPP2 (AR)	Evans syndrome (immune thrombocytopenic purpura and autoimmune hemolytic anemia), progressive Leukopenia, mild viral infections, mild developmental delay	Normal or slightly low CD4+ Tc counts	Senescent CD8+ Tc (impaired proliferation, enhanced staurosporine-induced apoptosis)	Premature immunosenescence (Tc and Bc and antinuclear antibodies), normal IgA and IgE levels, IgG and IgM levels high	[18,24]
Profuse warts	CARMIL2 (AR)	Recurrent bacterial, fungal and mycobacterial infections, molluscum contagiosum, EBV lymphoproliferative syndrome and other malignancy, atopy	Low Tregs, high frequency of naïve CD4+, but normal CD4+ overall	Poor Tc dependent antibody response Poor Tc function	Low frequency of memory B cells Ig normal or low	[17,25]
Warts	*IKBKG*/NFκB essential modulator (XL)	Opportunistic Infections: *P. jirovecii,* common, NTM, histoplasma, HSV, CMV, MCV infections	Tc normal or low	TCR activation impaired	Low memory and isotype switched Bc, monocyte dysfunction, low IgG, some elevated IgG, IgM	[18,20,24]
Tree man syndrome or common warts	CD28 (AR)	None	Low Tregs, Low central memory CD4 and CD8 T cells	Abolished CD28 costimulation response, impaired T cell proliferation upon antigens stimulation	Low NK cells	[7]

IEI, inborn errors of immunity; AR, autosomal recessive; XL, X-linked; Tc, T cells, Tregs, T regulators; EV, epidermodysplasia verruciformis; HSV, herpes simplex virus; VZV, varicella zoster virus; DOCK8, dedicator of cytokinesis 8; STK4, serine/threonine protein kinase 4; EBV, Epstein–Barr virus; NTM, nontuberculous mycobacteria; HSV, herpes simplex virus; CMV, cytomegalovirus; MCV, molluscum contagiosum virus; CARMIL2: capping protein regulator and myosin 1 linker 2; RHOH, Ras homolog family member H; IFNγ, interferon γ; TNFα, tumor necrosis factor α; NF-κB, nuclear factor kappa B, IKBKG, inhibitor of nuclear factor kappa B kinase regulatory subunit gamma.

**Table 3:** Etiologies, clinical phenotypes, and immunological phenotypes of warts associated with several impaired leukocyte subsets.

Disease Name	Gene/Protein (Mode of Inheritance)	Other Clinical Phenotypes	T Cell Counts	T Function	Other Immunological Features	Reference
WHIM syndrome	CXCR4 gof (AD)	Warts, genital dysplasia pneumonia, cellulitis, sinusitis, urinary tract infection, thrombophlebitis, omphalitis, osteomyelitis, soft tissue abscesses, HSV infections, VZV infections	Low	Low LPA/LPM Cutaneous anergy	Low IgG and IgA, normal antibody responses neutropenia low Bc: - Low CD27+ Bc - Low IgD+ and IgD-Bc	[26]
MonoMac syndrome DCML Emberger syndrome or WILD syndrome	GATA2 (AD)	Warts, susceptibility to mycobacteria, histoplasmosis, lymphedema, pulmonary alveolar proteinosis, myelodysplasia	Variable, Low Tc	Variable, impaired T cell proliferation upon mitogen stimulation	Monocytopenia, Low Bc Low NK	[18,24]
LAD syndrome	LAD1/ITGB2 (AR)	Warts, delayed cord separation with omphalitis, no pus formation, lack in inflammation is observed in infection area, periodontitis	Leukocytosis	-	Low CD18+ neutropenia	[18,20,24]
Warts	CD154/CD40L, tumor necrosis factor surface family 5 (XL)	*P. jirovecii* pneumonia, chronic watery diarrhea due to infection with cryptosporidium, liver and biliary tract disease with sclerosing cholangitis due to cryptosporidium parvum, and infections with hepatitis B and C viruses as well as CMV can result in liver and biliary tract tumors	Variable	Defect in CD40L production	Neutropenia, autoimmunity with immune TNFSF5 thrombocytopenia, hemolytic anemia, and immune mediated nephritis	[18,20]
Syndromic EV	DCLRE1C/ ARTEMIS (hypomorphic, AR)	Recurrent respiratory and gastrointestinal infections	Low CD4+ Tc	Impaired proliferative response and reduced counts of naïve T cells, restricted T cell receptor repertoire	Low B cell numbers and serum IgA levels increased sensitivity to ionizing radiation of fibroblasts	[18,27]
Warts	SPINK5 (AR)	Congenital icthyosis, bamboo hair, atopic diathesis, bacterial infections	Normal T cell counts, the proportion of naïve CD4^+^ T cells is reduced and the proportion of CD8^+^ T central memory elevated	-	Switched and non-switched Bc are reduced hyper IgE and IgA Other Ig: variably decreased impaired NK cytotoxicity	[18,20,28]
Warts	ADA (XL)	Chondrosternal dysplasia, deafness, pulmonary alveolar proteinosis, cognitive defects	Low Tc	-	Low Bc absent or reduced ADA activity (<1% of normal)	[18,20,24]

IEI, inborn errors of immunity; AR, autosomal recessive; AD, autosomal dominant; XL, X-linked; Tc, T cell; Bc, B cell; Treg, T regulators; NK, natural killers; LPA, lymphocyte proliferation to antigen; LPM, lymphocyte proliferation to mitogen; gof, gain of function; CXCR4, C-X-C motif chemokine receptor 4; WHIM, warts, hypogammaglobulinemia, infections, and myelokathexis; LAD-1, leukocyte adhesion deficiency type, ADA, adenosine demaminase; MAC, *Mycobacterium avium* complex; MonoMAC, monocytopenia *M. avium* complex infection; DCML, dendritic cell, monocyte, B cell, and NK cell lymphopenia; NTM, nontuberculous mycobacteria; SPINK5, serine protease inhibitor Kazal type 5; WILD syndrome: Warts, immunodeficiency, lymphedema, and dysplasia syndrome; DCLRE1C, DNA cross-link repair 1C; *ITGB2*, integrin beta chain β2.

## Data Availability

Not applicable.
